# Ustekinumab in the Treatment of Inflammatory Bowel Diseases: Evolving Paradigms

**DOI:** 10.3390/jcm13051519

**Published:** 2024-03-06

**Authors:** Giammarco Mocci, Antonio Tursi, Francesca Maria Onidi, Paolo Usai-Satta, Giovanni Mario Pes, Maria Pina Dore

**Affiliations:** 1 Division of Gastroenterology, “Brotzu” Hospital, 09124 Cagliari, Italy; giammarco.mocci@aob.it (G.M.); francescamariaonidi@aob.it (F.M.O.); paolousai@aob.it (P.U.-S.); 2 Territorial Gastroenterology Service, ASL BAT, 76123 Andria, Italy; antotursi@tiscali.it; 3 Department of Medical and Surgical Sciences, School of Medicine, Catholic University, 00168 Rome, Italy; 4 Department of Medicine, Surgery and Pharmacy, University of Sassari, 07100 Sassari, Italy; gmpes@uniss.it; 5 Baylor College of Medicine, One Baylor Plaza Blvd., Houston, TX 77030, USA

**Keywords:** Crohn’s disease, inflammatory bowel diseases, mucosal healing, safety, remission, ulcerative colitis, ustekinumab

## Abstract

Inflammatory bowel diseases, comprising Crohn’s disease (CD) and ulcerative colitis (UC), are chronic, relapsing, and remitting immune-mediated inflammatory diseases affecting the gastrointestinal tract. Ustekinumab (UST) is a monoclonal antibody that blocks the p40 subunit of the anti-interleukin (IL) 12/23. Pivotal trials (CERTIFI and UNITI-IM for CD, UNIFI for UC) established the efficacy of UST for the induction and maintenance of remission in both CD and UC, with the most favorable results in naïve patients to biologics. In recent years, a wealth of ‘real-world’ data has emerged supporting positive clinical, endoscopic, and histological outcomes in patients treated with UST, as well as reassuring safety data. More recently, the results of the first head-to-head trials of UST and tumor necrosis factor (TNF) antagonists were reported. Moreover, a number of studies exploring the role of UST in specific clinical settings, such as perianal CD, postoperative complications and recurrence, extraintestinal manifestations, chronic antibiotic-refractory pouchitis, and pregnancy, were reported. This review explores the results reported to date on UST, including those from pivotal trials, real-world data, and emerging studies regarding therapeutic drug monitoring and immunogenicity. The safety profile of UST was also reviewed.

## 1. Introduction

Ulcerative colitis (UC) and Crohn’s disease (CD) are the two most common forms of inflammatory bowel disease (IBD). The IBDs are commonly observed in the Western world and are caused by a complex interaction between genetic and environmental factors [1]. Both diseases are characterized by a relapsing and remitting course, and aggressive treatment is frequently required to prevent complications [2].

After discovering the critical pathogenetic role of tumor necrosis factor-α (TNF-α) in IBD, monoclonal anti-TNF-α antibodies were developed and successfully adopted in clinical practice. However, a number of patients do not respond or experience a secondary loss of response or intolerance to anti-TNF-α treatment. Therefore, novel therapeutic agents targeting alternative pathogenetic pathways were investigated and approved for IBD treatment [3,4,5,6].

Ustekinumab (UST) is a monoclonal antibody that blocks the p40 subunit of interleukin (IL)-12 and IL-23 [7], showing significant efficacy and safety in treating psoriatic arthritis [8]. Given the similarities in pathogenic mechanisms underlying IBD and chronic arthritis, UST was adopted in the therapy of IBD.

The IL-12 family comprises heterodimeric cytokines, including IL-12, IL-23, IL-27, and IL-35. Despite their similar structural characteristics, these molecules perform different functions. The cytokines IL-12 and IL-23, released in response to intestinal pathogens, have an essentially proinflammatory action, which is necessary for the differentiation of naïve CD4+ T cells [9]. Following binding to the receptors IL-12Rb1 and IL-12Rb2, the JAK/STAT pathway is activated, and in turn, the transducer proteins JAK2 and TYK2: more specifically, in response to IL-12, the protein STAT4 is phosphorylated, while in response to IL-23, STAT3, and STAT4 are phosphorylated [10]. The IL-12 drives cell-mediated immunity by activating T-cell proliferation using Th1 cells [11,12]. This cytokine has a heterodimer structure composed of p40 and p35 protein subunits, which, in turn, bind to a heterodimeric receptor complex consisting of IL-12 receptor (IL-12Rb1) and IL-12Rb2 chains expressed on the surface of T-cells and natural killer (NK) cells [13]. IL-23 plays a major role in the expansion of committed Th17 cells [10,14]. This cytokine is also heterodimeric with p40 (common to both IL-12 and IL-23) and p19 protein subunits [15]. The activation of this specific signaling pathway determines the expansion of committed Th17 cells [14]. Therefore, a blockade of the IL-12/23 system, using a specific antibody, inhibits Th1- and Th17-mediated adaptive immune responses, which play a fundamental role in the pathogenesis of CD [16], as well as UC [17].

**Figure 1:**
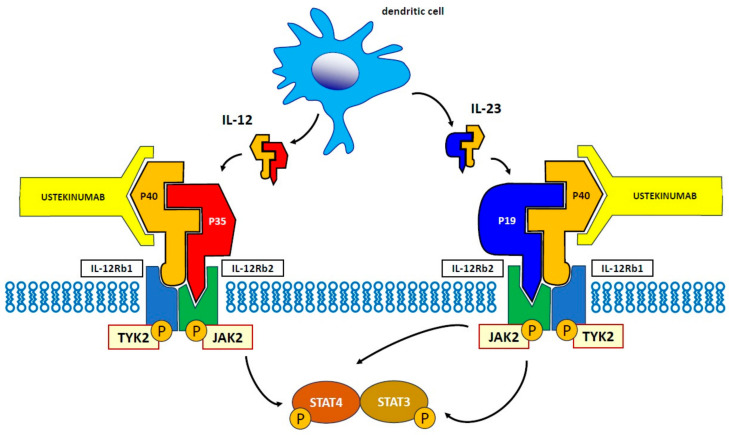
Mechanism of action of ustekinumab. For explanation, see the Introduction.

More specifically, IL-12 induces the differentiation of a naïve cluster of CD4+ T-cells into Th1 cells, releasing interferon (IFN)-γ [9,13]; whereas IL-23 induces the differentiation of naïve CD4+ T-cells into Th17 cells, which results in the activation of various proinflammatory cytokines, such as IL-6, IL-17, and TNF-α [18]. IL-23 is also capable of triggering a strong proinflammatory response through the activation of various immunocompetent cells, including CD8+ T-cells, innate lymphoid cells, γ/δ T-cells, and NK cells [19] (Figure 1).

Genome-wide association studies (GWAS) have identified IL-12 and IL-23 as the key cytokines in the pathogenesis of IBD [20]; therefore, therapies targeting the IL-12/IL-23 pathways have emerged and are in development.

Ustekinumab is a fully human IgG1k monoclonal antibody that binds to the p40 subunit of IL-12 and IL-23, inhibiting the interaction with the IL-12 receptor and subsequently blocking IL-12 signaling and further activation of the subset of Th1 cells. Moreover, it interferes with the IL-23-mediated immune response and, consequently, the activation of the Th17 cell subset [7].

Long-term data from the CERTIFI and UNITI-IM for CD, and UNIFI for UC, have proven the efficacy of UST for the induction and maintenance of remission in both IBDs, and the most favorable results were observed in naïve patients to biological drugs [21,22,23,24,25]. Numerous “real world” (RW) data that have emerged in recent years have confirmed the favorable clinical, endoscopic, and histological results in patients treated with UST, the safety of which has thus been strengthened [26,27,28,29,30,31,32,33,34,35,36,37,38,39,40,41,42,43,44,45,46,47,48,49,50,51,52,53,54,55,56,57,58,59]. The results of the first comparative study between UST and an anti-TNF-α have further supported this claim [60]. There are also specific studies that have investigated the role of UST in particular clinical situations, such as perianal CD [61,62,63], postoperative complications and recurrence [64,65,66], extraintestinal manifestations (EIMs) [67], chronic antibiotic-refractory pouchitis [68,69], and pregnancy.

This review aims to summarize the current literature regarding the mechanism of UST action, data from registrational trials on safety and efficacy, including open-label extension (OLE), observational and emerging RW evidence on its effectiveness in the treatment of IBD, and evolving paradigms with UST.

## 2. Materials and Methods

A thorough literature search was conducted using PubMed to identify all relevant articles published until November 2023. For the purpose of the present review, a comprehensive search was conducted by accessing multiple databases of the published literature, using the following search terms: “inflammatory bowel disease”, “Crohn’s disease”, and “Ulcerative colitis” matched with each of the following keywords: “Ustekinumab”, “Real life”, “Real world”. Additional studies were retrieved through the reference lists of detected articles and were independently and blinded examined by two authors (Mocci G and Tursi A), and any resulting discrepancy was discussed among all authors to reach a consensus. Subsequently, all detected sources, particularly RW studies with a significant number of patients, were analyzed and critically evaluated.

## 3. Results

### 3.1. UST in CD

#### Evidence from Randomized Controlled Trials (RCTs)

The favorable results obtained in the two phase-2 trials [21,22,23] paved the way for phase-3 trial programs, which were called UNITI. More in detail, two 8-week phase-3 induction trials (UNITI-1 and 2) and one 44-week phase-3 maintenance trial (IM-UNITI) were conducted [21,22,23]. The UNITI-1 trial included 741 patients who were non-responders or presented with unwanted side effects for anti-TNF-α. In contrast, in the UNITI-2 study, 628 patients were naïve to biologics or had been successfully treated with anti-TNF-α. In the IM-UNITI trial, approximately half (44%) of the patients received anti-TNF-α drugs [21,22,23]. In these studies, UST was administered at 130 mg or approximately 6 mg per kg i.v., while at 90 mg s.c. every 8 to 12 weeks to maintain remission.

In the UNITI-1, the primary endpoint of clinical response at week 6 (CDAI score decrease >100 points or <150 in patients with baseline CDAI 220–248) was observed in 33.7% of those dosed at 6 mg/kg and 34.3% of the 130 mg dose vs. 21.5% in placebo (*p* < 0.003 and 0.002, respectively). The secondary endpoint of clinical remission at week 8 (CDAI score <150 points) was observed in 20.9% of the 6 mg/kg group and 15.9% of the 130 mg group vs. 7.3% on placebo (*p* < 0.001, *p* = 0.003, respectively). Clinical response at week 8 (CDAI score 100-point response) was observed in 37.8% of the 6 mg/kg and 33.5% of the 130 mg UST groups, vs. 20.2% on the placebo group (each *p* < 0.001) [21,22,23]. In the UNITI-2 trial, the primary endpoint of clinical response at week 6 was achieved in 55.5% and 51.7% of the 6 mg/kg and 130 mg dosing groups, respectively, compared with 28.7% in the group who received placebo (*p* < 0.001 for both comparisons). The secondary endpoint of clinical remission and response at week 8 was achieved in a greater proportion of the 6 mg/kg and 130 mg UST dosing groups compared with placebo [21,22,23]. Patients who completed these induction trials could be recruited in the IM-UNITI study, in which 397 patients who responded to UST were randomly assigned to receive maintenance s.c. injections of 90 mg of UST (either every 8 weeks or every 12 weeks) or placebo. The IM-UNITI also enrolled 884 unrandomized patients. The primary endpoint for the maintenance trial was remission at week 44. Patients receiving 90 mg of UST every 8 weeks or every 12 weeks (53.1% and 48.8%, respectively) experienced significantly higher remission rates compared with the placebo arm (35.9%; *p* = 0.005 vs. UST every 8 weeks and *p* = 0.04 vs. UST every 12 weeks).

The ability of continued treatment with UST s.c. to maintain clinical response and remission over three years was investigated by another study (UNITI Long-Term Extension, UNITI-LTE). The IM-UNITI trial found that 38.0% of UST induction responders who were treated with the drug every 12 weeks and 43.0% of those who received the drug every 8 weeks were in remission at week 152 [22]. Lastly, 34.4% of patients in the every-8-weeks group and 28.7% in the every-12-weeks group were in clinical remission at week 252 [23].

The SEAVUE is the first clinical trial to directly and prospectively compare two approved biological treatments for CD, UST, and adalimumab (ADA) in a randomized, double-blind, treat-through design [60].

In this study, 386 patients who had failed conventional therapy and were biologic naïve, were randomized to ADA or UST induction followed by maintenance therapy with a primary endpoint of clinical remission at week 52 (CDAI score < 150). There was no significant difference in the proportion of patients in clinical remission between UST and ADA-treated patients (65% vs. 61%, *p* = 0.42). ADA-treated patients had higher rates of anti-drug antibodies compared to UST-treated patients (74% vs. 2%); however, the presence of anti-drug antibodies did not modify the treatment response. Endoscopic remission, defined as SES-CD ≤ 3, at 52-week rates, was largely equivalent (31% with ADA vs. 29% with UST) regardless of baseline SES-CD score.

### 3.2. ‘Real-World’ Experience in CD

Real-world data provide greater insights into the effectiveness of therapy in a heterogeneous and more complex patient population representative of clinical practice. A growing body of evidence from RW data for UST provides reliable evidence of its effectiveness and safety. After the publication of the UNITI pivotal trials, a number of real-life studies from Europe, Asia, and the North and South Americas have confirmed UST efficacy in daily practice.

#### 3.2.1. UST for Bio-Experienced Patients

In most real-life studies, UST was used on anti-TNF-α failure or refractory patients [70,71]. In this specific clinical context, remission at 24 weeks and (when available) at 52 weeks ranged from 31% to 75% and 25% to 60%, respectively [26,27,28,29,30,31,32,33,34,35,36,37,38,39,40,41,42,43,44,45,46,47,48,49,50,51,52,53,54]. Moreover, mucosal healing (MH) and fecal calprotectin (FC) levels significantly improved under UST treatment [26,27,28,29,30,31,32,33,34,35,36,37,38,39,40,41,42,43,44,45,46,47,48,49,50,51,52,53,54]. Interestingly, the results comparison of studies conducted on different continents showed that UST acts similarly, namely between 24 and 52 weeks. The remission rate was about 25% in North America [32,34], 39% in South America [43], 25–60% in Europe [26,27,28,29,30,35,36,37,40,41,42,46,47,48], and 31–84% in Asia [31,38,39,45].

Two meta-analyses confirmed these results. In a systematic review and pooled analysis of RW evidence, Engel et al. found a pooled remission rate at week 24 of 39% (range 18–65%) [72]. Macaluso et al. observed a pooled remission rate of 34% (range 18–65%) at week 24 and of 40% at 52 weeks [73]. These results are superior to those obtained in pivotal trials, confirming a better response rate in RW studies.

In the absence of prospective RCTs comparing available treatments, some RW studies often tried to overcome this limit using the propensity score, a statistical method able to reduce the selection bias, to compare head-to-head UST vs. other biologics. Ahmed et al. found that UST was not superior to ADA at week 56 (27% vs. 25%, *p* = 0.820) [74]; However, Alric et al. found a higher response rate with UST than Vedolizumab (VDZ) at week 48 (54.4 vs. 38.8, *p* = 0.03) [75]. Instead, Lenti et al. did not observe the superiority of UST compared with VDZ at week 14 (*p* = 0.631) and week 52 (*p* = 0.157) [76]. Singh et al. compared UST vs. anti-TNF-α and VDZ in a large population of CD patients. The UST was better than TNF-α antagonists and VDZ in terms of response; however, differences in the hospitalization or surgery risk were not detected [77]. Onali et al. found UST slight but not significantly better than VDZ in obtaining clinical remission [78]. Finally, Kappelman et al. did not observe a difference in treatment persistence between UST and VDZ. Still, UST was associated with a lower rate of all-cause hospitalization (adjusted hazard ratio [aHR] 0.73), nonsurgical CD hospitalization (aHR 0.58), and hospitalization for infections (aHR 0.56) [79].

#### 3.2.2. UST for Bio-Naïve Patients

Real-world studies have also investigated UST in CD patients never exposed to biologics. Overall, the remission rate was higher in this setting.

In a retrospective, multicenter, multinational consortium of UST-treated CD patients, the authors observed a higher duration of remission at week 52 in bio-naïve compared with bio-experienced CD patients (55% vs. 40%, respectively). Importantly, an independent association was observed between previous exposure to anti-TNF-α (HR, 0.72) and VDZ (HR, 0.65) and a lower probability of responding to UST [80]. A study conducted in Belgium reported a 12-month remission of nearly 74% [81], and a recent Spanish study of CD reported remission at weeks 16 and 52 of 93% and 82%, respectively [82]. These findings were confirmed by a Canadian trial, which reported clinical remission in 59% of bio-experienced and 79% of bio-naïve CD patients [83]. In contrast, in a Brazilian trial, statistically significant differences were not observed in the remission rate recorded at one year between bio-experienced and bio-naïve CD patients treated with UST or VDZ (39.4 vs. 39.8: *p* = 0.96), respectively [45].

Finally, UST with other biologics was also compared in a number of RW studies. In a retrospective study conducted in two tertiary centers, Rivière et al. compared UST with anti-TNF-α drugs in bio-naïve CD patients with luminal disease. The results showed that anti-TNF-α as a first-line treatment was more efficient than UST at three months (*p* = 0.02), whereas no difference was detected during a 40-month follow-up (*p* = 0.29) [84]. These findings were probably linked to the effect of IFX rather than ADA. Zhdanava et al. reported that, at 12 months, bio-naïve UST-treated CD patients showed a significantly higher treatment persistence rate than ADA-treated CD patients [85]. On the other hand, a Belgian study was unable to detect differences in the clinical remission rate between ADA and UST bio-naïve CD patients at week 26 (adjusted odd ratio [aOR] 1.30; *p* = 0.72) and at week 52 (aOR, 1.60; *p* = 0.41) [86].

### 3.3. UST in UC

#### 3.3.1. Evidence from Randomized Controlled Trials

More recently, UST was approved by the European Medicines Agency and the Food and Drugs Administration to treat moderate-to-severe active UC patients with inadequate response, lost response to, or intolerance to either conventional therapy or biologic, or with medical contraindications to such therapies. UST efficacy and safety were investigated in a double-blind, randomized phase-3 trial (UNIFI) among patients with moderate-to-severe active UC. This study consisted of a single protocol combining 8 weeks of induction therapy and 44 weeks of maintenance therapy, accounting for a total of 52 weeks of therapy [24]. In this study, adult patients were recruited with moderate-to-severely active UC (defined as a Full Mayo Score ranging from 6 to 12, with a minimum endoscopic subscore of 2) and a medical history of inadequate benefit/intolerance to conventional or biological drugs or both. According to the endpoints, induction, and maintenance were analyzed. In the ongoing long-term extension study, participants will continue with the same treatment regimen received at the end of the maintenance study [24].

At baseline, 961 patients were randomly assigned to either a single i.v. infusion of 130 mg of UST, a dose based on the weight that approximated to 6 mg/kg (260 mg, weight ≤ 55 kg; 390 mg, weight > 55 kg and ≤85 kg; 520 mg, weight > 85 kg), or placebo. Overall, nearly 48.0% of patients had previously failed biological therapies (13.4% both anti-TNF-α drugs and VDZ), and 51% of them were on concomitant steroids at baseline.

UST i.v. was more effective than placebo (15.6% vs. 5.3%) for inducing clinical remission in patients at week 8. Week 8 clinical responders were re-randomized into three different maintenance arms: 90 mg UST s.c. every 12 weeks (q12w), q8w, or placebo. UST s.c. q12w or q8w was more effective than placebo (38.4% or 43.8% vs. 24%) for maintaining clinical remission in responders at week 44 from induction. No significant differences were observed in patients with or without previous treatment failure with biologics [24].

After completing the maintenance phase, patients who received UST entered the long-term extension study until week 220, maintaining the same treatment regimens. At week 200, 55.2% were in symptomatic remission, with a greater proportion of biologically naïve patients (67.2%, 117/174) than those with a history of biological failure (41.6%, 67/161). Finally, among patients in symptomatic remission at week 200, 96.4% were corticosteroid-free [87].

#### 3.3.2. ‘Real-World’ Experience in UC

More recently, after the approval of UST for UC, some retrospective, observational studies, including unselected patients, have evaluated the effectiveness and safety of UST for UC in clinical practice [49,50,51,52,53,54,55,56,57,58,59,88]. Overall, although limited by the small sample sizes enrolled and the short follow-up time, these studies provided further reliable evidence for the effectiveness and safety of UST. A systematic review of 13 RW studies [89] noted that clinical remission and induction were achieved in 24% to 61% and in 47% to 77% of patients, respectively. Moreover, clinical remission was achieved in 33% to 79% of cases at the 52-week follow-up, whereas steroid-free remission was reported in six studies and ranged from 14% to 67%. Finally, lack of effectiveness, refractory disease, and loss of response were the main reasons for UST discontinuation. The heterogeneity in the results could be explained by a number of reasons. For instance, retrospective studies generally lack controls for some variables, or consist of small cohorts. In addition, as underlined by Macaluso et al., the outcomes are not well defined or are based on doctors’ judgment taking into consideration subjective and not objective parameters, such as CRP or fecal calprotectin [90].

According to Hong et al., negative factors that might predict clinical remission and response with UST include a history of TNF-α antagonist primary nonresponse and a baseline Mayo endoscopic score of 3, at three months [53]. Chaparro et al. showed that CRP serum concentration over the upper limit of the normal range was the only factor significantly associated with a lower probability of achieving remission [49].

A meta-analysis of RW studies included a total of 19 studies, with 3786 patients, of which >92% were previously treated with any biologic, 61.1% with both anti-TNF-α and VDZ, and 16.4% with any biologic and tofacitinib [91]. Among the UC patients, 45.4% were in clinical remission by weeks 8, 43.8% (38.4–49.2%) by weeks 12–16, 44.6% by month 6, and 50.6% by month 12. Overall, 58.2% of patients displayed endoscopic improvement by month 12. Clinical response was achieved in 61.2%, 59.4%, 65.2%, and 76.8% by week 8, weeks 12–16, month 6, and month 12, respectively. Corticosteroid-free remission was achieved in 18.7%, 36.8%, 34.5%, and 39% at week 8, weeks 12–16, month 6, and month 12, respectively. Interestingly, almost 30% of the patients needed dose escalation, which was effective in 40% of them. However, this meta-analysis suffers from several biases, including high (>80%) heterogeneity. Parakkal et al. [55] and Honap et al. [54] reported remission rates of 23.9% and 20% at week 8 and 40.4% and 43.6% at week 26, respectively.

### 3.4. Safety

In various studies, UST has shown a good safety profile in the treatment of patients with CD. For example, in the pivotal studies of UNITI-1 and -2, the prevalence of adverse events (AEs) was (65.9% and 55.6%), not significantly different from placebo (64.9% and 54.3%). Even with regard to serious AEs, prevalences were similar among patients treated with UST (7.2% and 2.9%) compared to placebo (6.1% and 5.8%). In agreement with previous studies, in IM-UNITI the AEs in general, as well as the severe ones, measured per 100 patient-years of follow-up from week 0 to week 156 at different doses of UST, were even lower compared to placebo (389.70 vs. 444.17) and (18.97 vs. 19.54), although without statistical significance. The occurrence of serious infections was similar in the two groups (4.21 vs. 3.97) [21,22,23].

In the trial conducted by the UNIFI study group in patients with moderate-to-severe UC, the AEs in the groups exposed to 130 mg of UST, 6 mg of UST/kg, or placebo were comparable (41.4%, 50.6%, and 48.0%, respectively) and the total patients who complained of at least one serious AE were 3.7%, 3.4%, and 6.9%, respectively.

No deaths occurred among the primary population in the maintenance study. The infection rates were similar across the treatment groups, and serious infections were infrequent: 1.7% in the q8w group; 3.5% in the q12w group; and 2.3% with placebo. AEs led to discontinuation of UST in 20 patients in the placebo group; five and nine in the q8w and q12w groups, respectively [24].

The good safety profile of UST was also confirmed in real-life studies. Patients with CD, treated with UST, reported a mean percentage of about 11% for AEs, although the majority were mild and did not require treatment withdrawal. However, in a retrospective study from the US performed in patients with complex perianal disease (100% previously exposed to ≥2 anti-TNF-α, 61.3% prior perianal surgery, and 36.2% taking concurrent immunomodulators) treated with UST, the authors reported a rate of about 44% with AEs [62].

In UC, a systematic review of RW data found that patients with AEs ranged from 2.6% to 32% [89]. In another recent systematic review with a meta-analysis of RW, the incidence rates (iRs) of colectomy, mild and severe AEs, and serious infections were 4.8, 7.9, 0.8, and 0.3 per 100 patient-years, respectively [91]. The most common non-infectious AE, except IBD exacerbation, was arthralgia (1.94%), followed by a skin rash (1.55%).

### 3.5. UST in Special Situations

#### 3.5.1. UST for Perianal Disease

UST was also evaluated in fistulizing perianal disease but with modest results. A Network Meta-Analysis of RCTs aimed to compare the efficacy of biologic therapies in inducing response and remission in fistulizing CD, showed the superiority of UST to placebo in inducing a response (OR, 0.48; 95% CI, 0.26–0.860). However, the drug was ineffective in inducing remission (OR, 0.50; 95% CI, 0.13–1.93) [92].

The efficacy of UST in perianal CD was assessed in detail by the *Groupe d’Étude Thérapeutique des Affections Inflammatoires du Tube Digestif* in a national multicenter retrospective cohort study. Among 207 patients collected for the analysis, 99% were previously exposed to at least one anti-TNF-α, and 28% of them also received VDZ. The treatment was successful in 38.5% of cases with active perianal disease; moreover, among 88 patients with a seton at the time of starting treatment, 29 (33%) were able to remove it [61]. In a retrospective cohort, Godoy Brewer et al. found that, at 6 and 12 months, 48.1% and 55.6% of patients had an improved fistula response, but none achieved fistula healing [62]. Finally, according to the study of Yao et al., radiological healing of perianal fistulas was obtained in 44.8% of patients by using UST [63]. Overall, the few RW studies in the literature on UST for perianal disease reported modest effectiveness.

#### 3.5.2. UST for Postoperative Recurrence

No treatment has been approved in CD after ileocolonic resection, despite the risk of postoperative recurrence. In the presence of at least one of the clinical risk factors, the current ECCO guidelines recommend prophylactic immunosuppressive therapy with thiopurines or anti-TNF-α agents to prevent postoperative recurrence [93]. Few RW studies are currently available in this specific setting of CD patients. Tursi et al., in a preliminary, retrospective study, reported that in 73.3% of CD patients refractory to anti-TNF-α, the use of UST obtained MH, defined as a Rutgeerts’ score < 2 [65]. A prospective and comparative study showed a rate of endoscopic postoperative recurrence of 42% for UST and 40% for VDZ in a cohort of patients treated with anti-TNF-α drugs [64]. Finally, in an Italian study, Macaluso et al. confirmed the promising role of UST in preventing postoperative recurrence. After an average interval of 14.5 ± 5.5 months following initiation of UST, the authors observed that 50% of patients experienced endoscopic healing [66].

#### 3.5.3. UST for Extraintestinal Manifestations

The occurrence of extraintestinal manifestations (EIMs) during IBD is challenging. Anti-TNF-α agents are well-studied and validated in this field [94]. The use of alternative biologics, such as UST, remains unclear, although Guillo et al. found that UST could be an effective option for the treatment of EIMs of CD. UST treatment was shown to be highly effective for arthralgia, psoriasis, psoriatic arthritis, pyoderma gangrenosum, and erythema nodosum, whereas no efficacy was found in axial spondyloarthritis [67]. An exciting study assessed the effect of UST on EIMs in patients who failed to respond to anti-TNF-α agents. Twenty-four CD patients with EIMs including articular disease, rheumatoid arthritis, seronegative arthritis, ankylosing spondylarthritis, erythema nodosum, uveitis, sclerosing cholangitis, and hidradenitis suppurativa treated with UST reported a significant improvement/remission rate [95].

A retrospective cohort study including IBD adult patients treated either with VDZ and exposed to UST and with EIMs before treatment initiation showed a clinical response of EIMs at week 52 in 36% (18/50) [96]. In addition, the safety profile and efficacy in EIMs suggest that UST may be a reasonable candidate for a combination of targeted therapies, for drug-refractory IBD patients without other medical alternatives, as well as for those with concomitant EIMs [97,98,99]. All these specific clinical contexts will require further, larger, and longer studies.

#### 3.5.4. UST for Pouchitis

Restorative proctocolectomy with ileal pouch-anal anastomosis (IPAA) is routinely performed in patients with UC undergoing colectomy [100,101]. Idiopathic inflammation of the pouch—referred to as pouchitis—is the most common long-term complication of IPAA [102,103]; it develops in approximately half of patients within 5 years after surgery [104] and recurs in more than half of the affected patients.

The conventional treatment for confirmed pouchitis consisted of antibiotics such as ciprofloxacin and metronidazole [102]. Up to 15% of patients, however, develop chronic pouchitis and become dependent on antibiotics for symptom relief, although in some cases, despite chronic antibiotic therapy, they complain of continuous symptoms [102,105,106,107]. Chronic antibiotic-refractory pouchitis represents an indication for biological therapy. A meta-analysis including 313 patients with inflammatory complications of the pouch after IPAA for UC evaluated the role of infliximab or ADA in clinical remission. The anti-TNF-α drugs were able to achieve in the short- and long-term (12 months) remission in about 50% of patients [107]. Other treatments are usually recommended for refractory patients or those with specific contraindications to anti-TNF-α drugs [108]. The real-life data for the use of UST in this setting are still scarce and limited by small sample sizes and large heterogeneity of therapy protocols/outcome definitions [9,10,11,12,13,14,15,16,17,18,19,20,21,35,45,47,48,55,61,62,63,68,69,70,71,72,74,75,76,77,78,79,80,81,82,83,84,85,86,87,88,89,91,92,93,94,95,96,97,98,99,100,101,102,103,104,105,106,107,108,109,110,111].

In a retrospective, single-center study, 24 patients with chronic antibiotic (ciprofloxacin or metronidazole)-refractory pouchitis and previously treated with biologics different from UST (50%) or immunomodulators (25%) were evaluated. After other pouch disorders were ruled out, patients received a UST 90 mg i.v. loading dose infusion, followed by 90 mg s.c. injections q8w [68]. After a median follow-up of 12.9 months, clinical remission was obtained in half of the patients based on the physician’s judgment and according to the number of bowel movements per day. Pouchoscopies post-UST treatment (median time 7.4 months) were available from 13 patients and a decrease in the median Pouchitis Disease Activity Index from baseline (5 to 4, *p* = 0.016) was observed.

Dalal et al. reported on a cohort of 46 patients treated with UST with mixed pouch disorders, six chronic antibiotic-refractory pouchitis, four cuffitis, and 36 CD of the pouch [109]. All patients were previously exposed to anti-TNF-α, and 24 also to VDZ.

According to the physician’s judgment, a clinical response at 8–16 weeks was observed in 80.4% of patients. Dose intensification to q6w or q4w was required in 23 patients after a median of 223 days, and a clinical response was obtained in 60.8% of them within the subsequent 8–16 weeks. Younger ages at both UC diagnosis (HR = 0.94, 95% CI = 0.90–0.99) and UST start (HR = 0.96, 95% CI = 0.92–0.99) were associated with a shorter time to dose intensification.

In a systematic review aimed to evaluate the efficacy of UST in chronic refractory pouchitis [69], clinical response and clinical remission were observed in 63% and 10% of patients, respectively, after 4–12 weeks, whereas an endoscopic response was reported in 60% of patients with chronic pouchitis after 24–32 weeks of treatment. Small sample sizes and large heterogeneity of therapy protocols/outcome definitions were significant limitations of these studies.

In conclusion, there is a limited and inconclusive body of evidence suggesting UST as a therapeutic option for patients with chronic pouchitis and CD of the pouch refractory to other therapies.

#### 3.5.5. UST in Pregnancy

Similarly to anti-TNF-α drugs, UST is also an IgG1 able to cross the placenta through the neonatal Fc receptor (FcRn). In the first phase of gestation, low levels of IgG antibodies from the maternal serum may pass over the placental tissue by passive diffusion [112]. Starting from the 13th to the 17th week of pregnancy and following, the passage of IgG from the mother to the fetus through the placenta structures, such as neonatal FcRn, as well as other placental Fcγ receptors (FcγR), increases considerably [113].

UST drug levels appear stable in pregnancy, with a delivery infant:maternal ratio similar to that of anti-TNF–α drugs, and infant UST clearance was complete by 20 weeks post-partum [114]. Considering that biologic drugs are IgG1 monoclonal antibodies, secretion into the breast milk is usually minimal. Accordingly, low levels (max 1.57 μg/mL) of UST were detected in the breast milk of mothers exposed to this drug [115].

A favorable safety profile of UST for pregnant women was initially observed among patients treated for psoriatic diseases [116]. Few data have been reported up to now for females affected by IBD, who conventionally receive higher doses compared with the dermatological and rheumatological indications. Although UST has not been extensively studied in pregnant women with IBD, existing data suggest favorable pregnancy and neonatal outcomes that were comparable with those in patients treated with anti-TNF–α or other therapies. Cases have been reported of mothers exposed to UST during pregnancy and lactation without observable negative consequences for them or their children [117,118]. The effects of UST in pregnant women with IBD were also evaluated in the PREGNANCY-GETAID study, without negative effects on maternal or neonatal outcomes. Among 29 pregnancies, 26 (90%) resulted in live births, two (7%) in spontaneous abortions during the first trimester, and one (3%) in elective termination. Mild maternal complications were reported in two patients. Rates of prematurity, spontaneous abortion, congenital malformations, and maternal complications were comparable between the UST and anti-TNF-α groups [119]. In a prospective, multicenter, observational study performed on consecutive females with IBD exposed to UST or VDZ for two months before conception or during pregnancy, 43 out of 54 (79.9%) pregnancies exposed to UST resulted in live births, and 11 (20.4%) led to spontaneous abortion, which was not significantly different from the control group [119]. These data were confirmed by the preliminary results from the DUMBO prospective registry [120].

The Pregnancy in Inflammatory Bowel Disease and Neonatal Outcomes (PIANO) is a national study of females with IBD and their children aimed at evaluating the safety of IBD medications on pregnancy and on short- and long-term outcomes in children [121]. Outcomes measured in the PIANO registry include spontaneous abortion, preterm birth, small for gestational age birth weight, low birth weight, intrauterine growth restriction, cesarean section, and requirement for neonatal intensive care at birth, placental disorders, congenital malformations, and finally, infant infections. Questionnaires were administered at the study baseline, throughout pregnancy, and after delivery. Patients were stratified into two cohorts: (i) an IBD cohort exposed to VDZ, UST, anti-TNF–α agents, thiopurines, or a combination of these agents, and (ii) a control cohort not exposed to these drugs. Among the 1669 patients with completed pregnancies, there were 1610 live births. The maternal mean age was 32.1 (SD 4.6) years at delivery and 47 of them were exposed to UST. Between the UST-treated and the control cohorts, differences among the variables included in the study were not observed [120]. Outcome data following maternal use of UST during pregnancy are becoming available from registries and prospective studies [122,123,124,125,126,127]. Based on these data, in the recent ECCO guidelines, the authors concluded that no increased risk of adverse pregnancy outcomes was identified with UST [2,126,127,128]. However, the overall study population is still too small to drive any firm conclusions, and there is a need for further evidence to support these findings. Furthermore, due to limited data, careful follow-up of pregnant patients treated with UST is advised.

## 4. Conclusions

UST is a much-needed addition to the increasing armamentarium in the treatment of IBD, and its effectiveness in patients with other immune-mediated diseases, such as psoriasis and psoriatic arthritis, is particularly appealing. UST has shown efficacy both as a first-line and second-line drug for the induction and maintenance of remission in IBD. Real-world data have supported its effectiveness and safety profile. Recent evidence on pregnancy, prevention of postoperative recurrence, fistulizing CD, and pouchitis appear promising.

## Data Availability

The data supporting the conclusions of this article are provided within the article and are available from the corresponding authors upon request.
